# Combined treatment with quercetin and imperatorin as a potent strategy for killing HeLa and Hep-2 cells

**DOI:** 10.1007/s11010-014-2032-4

**Published:** 2014-03-30

**Authors:** Dorota Bądziul, Joanna Jakubowicz-Gil, Roman Paduch, Kazimierz Głowniak, Antoni Gawron

**Affiliations:** 1 Department of Comparative Anatomy and Anthropology, Institute of Biology, Maria Curie-Skłodowska University, Akademicka 19, 20-033 Lublin, Poland; 2 Department of Immunology and Virology, Institute of Microbiology and Biotechnology, Maria Curie-Skłodowska University, Akademicka 19, 20-033 Lublin, Poland; 3 Department of Pharmacognosy with Medical Plant Unit, Medical University, Chodźki 1, 20-093 Lublin, Poland

**Keywords:** Cancer, Cell death, Imperatorin, Quercetin

## Abstract

The aim of the present study was to assess the effect of quercetin and imperatorin administered separately and in combination on apoptosis and autophagy induction in human cervical carcinoma HeLa cells and laryngeal carcinoma Hep-2 cells cultured in vitro. Conducted MTT measurements proved that quercetin and imperatorin displayed a strong antiproliferative activity manifested in markedly reduction of HeLa and Hep-2 cells viability as a result of treatment with 50 μM of each compound. Further cell staining assays revealed that concentration mentioned above generated the highest percentage of apoptotic cells especially in the case of application of both drugs for 48 h. Simultaneous quercetin and imperatorin administration induced apoptosis remarkably stronger than treatment with single drugs. Experiments at the molecular level confirmed these results accompanied with the decreased Hsp27 and Hsp72 expression and, in addition, with increased caspases activity. Autophagy was not observed and no significant changes in the expression of beclin-1 were noticed. Additionally, experiments were performed on the above-mentioned cell lines with blocked Hsp27 and Hsp72 expression. In these cells, no significant changes in the sensitivity to apoptosis induction upon quercetin and imperatorin treatment were observed. The present study has provided evidence supporting the potential of the combination of quercetin and imperatorin drugs as a novel tool to be used in anticancer therapy. Our results have also demonstrated that blocking of the Hsp27 and Hsp72 gene expression is not enough to sensitize cancer cells to programmed cell death induction in HeLa and Hep-2 cells.

## Introduction

In the past decade, the strategy for killing cancer cells through programmed cell death (apoptosis, autophagy) has been extensively studied. Literature data indicate a number of details concerning the regulation of both apoptosis and autophagy. Manipulation of these processes, as well as precise control of their pace and intensity may lead to the desired destination, being one of the strategies in the fight against cancer.

Apoptosis, also known as type I programmed cell death, is an essential physiological process playing a crucial role in tissue development and homeostasis. To date, research indicates that apoptotic signals may proceed through two main pathways, intrinsic (mitochondrial) related to cytochrome *c* release into the cytosol and extrinsic associated with the activation of death receptors. However, regardless of the type of apoptosis, both pathways lead to activation of caspases [1–5]. In turn, autophagy, i.e., type II programmed cell death, is a phylogenetically old process used as a tool not only for death but also for survival. Autophagy is known as an intracellular system of degradation of cytoplasm components in particular long-half-life proteins through lysosomal enzymes. The outcome of autophagy is always the same—total and irreversible dismantling of macromolecular substrates to their basic components [6–9].

Heat shock proteins have become the oldest cell protecting system; also called molecular chaperones, they are important effectors of cellular stress response. The scope of Hsps duties includes involvement in assistance with the native protein folding, maintenance of the proper conformation of multiprotein complexes, and degradation of senescent proteins in a situation where repair is not possible [10–12]. One of the best-studied proteins are Hsp27 and Hsp72, the most strongly and universally synthesized chaperones. Hsp27 and Hsp72 inhibit key effectors of the apoptotic machinery; therefore, accumulation of these proteins in the cell is an important cytoprotective factor allowing survival in adverse conditions not only in normal cells. Numerous investigations indicate overexpression of Hsp27 and Hsp72 observed in many types of cancer; hence, it is believed that they stimulate the process of carcinogenesis [13–15].

One of the well-known Hsps inhibitors is quercetin (3,3′,4′,5-7-pentahydroxyflavone), one of the best-described flavonoid. Quercetin, widely distributed in the plant kingdom, has become an ingredient of most daily-consumed fruit and vegetables. Like many compounds of this group, it has strong antioxidant, antiinflammatory, and antiproliferative properties. Recently quercetin has gained special attention as a potential anticancer agent inducing apoptosis in numerous types of cancer [16–20]. The mechanism of this reaction is based on inhibiting the activity of DNA topoisomerase I/II, modulation of signaling pathways, release of cytochrome *c*, and activation of caspases [21, 22].

Imperatorin (8-isopentenyloxypsoralen), a major active furanocoumarin isolated from the root of *Angelica officinalis*, has been reported to possess a wide range of biological activities including analgesic, antiinflammatory, anticoagulant, and photosensitizing properties. Recent reports have shown pharmacological actions of imperatorin against cancer manifested in suppression of oncogenes, inhibition of proliferation, arresting the cell cycle in the G1/S phase, and induction of apoptosis in several types of cancer [23–27].

Therefore, the aim of the present study was to examine the effect of quercetin and imperatorin administered separately or in combination on programmed cell death induction (apoptosis and autophagy) in the human cervical carcinoma HeLa and laryngeal carcinoma Hep-2 cell lines cultured in vitro. In order to determine the pathways through which apoptosis and autophagy take place in cell lines studied, we investigated the expression of marker proteins involved in these processes at the molecular level. We also assessed the potential of Hsp27 and Hsp72 expression in protecting cancer cells from cell death induction.

## Materials and methods

### Cells and culture conditions

The human cervical carcinoma cells (HeLa) and laryngeal carcinoma cells (Hep-2) were grown in a 1:1 mixture of DMEM and Nutrient mixture F-12 Ham (Ham’s F-12, Sigma) supplemented with 10 % FBS (Life Technologies, Karlsruhe, Germany), penicillin (100 μg/ml, Sigma), and streptomycin (100 μg/ml, Sigma). The cultures were kept at 37 °C in humidified atmosphere of 95 % air and 5 % CO_2_.

### Imperatorin isolation

Imperatorin was isolated from the fruits of *A.* *officinalis* in the Department of Pharmacognosy, Medical University of Lublin, Poland. The air-dried and powdered fruits of *A.* *officinalis* were extracted with petroleum ether exhaustively in the Soxhlet apparatus, which yielded a fraction of furanocoumarins obtained as a semi-crystalline sediment from the concentrated extract. Then, the imperatorin-rich sediment obtained from the fruits of *A.* *officinalis* was first dissolved in hot dichloromethane, and then subjected to crystallization with cold *n*-hexane. The sediment formed was recrystallized three times in methanol, which led to isolation of imperatorin. The identity and purity of imperatorin were confirmed by HPLC and H-NMR analyses [28].

### Drug treatment

Quercetin (Sigma) at the final concentrations 50 and 100 μM and imperatorin at the final concentrations 50 and 100 μM were used in the experiments. The drugs were dissolved in dimethyl sulfoxide (DMSO, Sigma). The final concentration of DMSO in the culture medium did not exceed 0.01 %, which as indicated in preliminary experiments, did not influence cell viability and the expression of the proteins studied. Three variants of drug treatment were performed. In the first one, HeLa and Hep-2 cells were incubated only with quercetin or only with imperatorin for 24 and 48 h. In the second, quercetin and imperatorin were added to the culture medium at the same time and incubated for 24 and 48 h. In the third variant, the cells were pre-incubated with quercetin or imperatorin for 6 or 24 h followed by the administration of the other compound and incubated for the next 18 or 24 h. Control cells were incubated with 0.01 % of DMSO.

### MTT assay

To determine HeLa and Hep-2 cells viability upon quercetin and imperatorin treatment the colometric MTT (3-(4,5-dimethylthiazole-2-yl)-2,5-diphenyltetrazolium bromide) metabolic activity assay was used. HeLa and Hep-2 cells grown in 96-well multiplates in 100 μl of culture medium supplemented with 2 % FBS were incubated for 3 h with MTT solution (5 mg/ml, 25 μl/well) (Sigma). The yellow tetrazolium salt was metabolized by viable cells to purple crystals of formazan. The crystals were next solubilized overnight in a mixture consisting of 10 % SDS (Sigma) in 0.01 M HCl. The final product of cell metabolism was quantified spectrophotometrically by absorbance measurement at 570 nm wavelength using an E-max Microplate Reader (Molecular Devices Corporation, Menlo Park, CA, USA).

### Detection of apoptosis and necrosis with fluorochromes

For identification of apoptosis and necrosis, the HeLa and Hep-2 cells were stained with a mixture of fluorescent dyes Hoechst 33342 (Sigma) and propidium iodide (PI) (Sigma), respectively [29]. The staining mixture was added in a volume of 2.5 μl/ml to the medium in which the cells were grown and incubated for 5 min at 37 °C in the dark. Apoptotic and necrotic cells were visualized and scored (at least 1,000 cells from randomly selected fields) under a fluorescent microscope (Nikon E800). Cells undergoing apoptosis demonstrated blue fluorescent nuclei (intact or fragmented) as a result of staining with Hoechst 33342. In turn cells stained with PI and exhibiting pink fluorescent nuclei were interpreted as necrotic. Three independent experiments were performed.

### Detection of autophagy with acridine orange

Autophagy is a process that involves sequestration and delivery of cytostolic components to the lysosome for degradation; it is characterized by formation and promotion of acidic vesicular organelles (AVOs). Vital staining with acridine orange was performed for identification of AVOs in HeLa and Hep-2 cells treated with quercetin and/or imperatorin [30]. The cells were incubated with the fluorochrome at a final concentration 1 μg/ml for 15 min in the dark. Autophagic positive cells demonstrated typical granular discretion of AVOs in the cytoplasm. Morphological analysis was performed under a fluorescent microscope (Nikon E800). Autophagic cells were visualized and counted (at least 1,000 cells from randomly selected fields). Three independent experiments were performed. The percentage of autophagic cells was calculated as the number of cells with AVOs versus the total number of stained cells. Each experiment was performed in triplicate.

### Immunoblotting technique

After the quercetin and/or imperatorin treatment, the HeLa and Hep-2 cells were lysed in hot SDS-loading buffer (125 mM Tris–HCl, pH 6.8; 4 % SDS; 10 % glycerol; 100 mM DTT), boiled in a water bath for 10 min, centrifuged at 10,000×*g* at 4 °C for 10 min, and the supernatants were collected. The Bradford method was used to determine the concentration of protein in the cell-free extracts obtained [31]. Samples of supernatants containing 80 μg of proteins were separated by 10 % SDS–polyacrylamide gel electrophoresis [32], and subsequently transferred onto the Immobilon P membrane (Millipore). Following the transfer, non-specific binding sites on the membrane were blocked with 3 % low fat milk in PBS for 1 h and incubated overnight with rabbit polyclonal anti-beclin-1 antibody (Sigma) diluted 1:1,000, goat anti-Hsp27 monoclonal antibody (Santa Cruz Biotechnology) diluted 1:1,000, and anti-Hsp72 (Santa Cruz Biotechnology) diluted 1:1,000. After the incubation, the membranes were washed three times for 10 min with PBS containing 0.05 % Triton X-100 (Sigma) and incubated for 2 h with a 1:30,000 dilution of alkaline phosphatase-conjugated anti-rabbit IgG or anti-goat IgG (Sigma). The membranes were visualized by the colorimetric reaction with alkaline phosphatase substrate (5-bromo-4-chloro-3-indolylphosphate and nitro-blue tetrazolium, Sigma) in a color development buffer (DMF, Sigma). Quantitative evaluation of the expression of heat shock proteins with molecular weights of 27 and 72 kDa and beclin-1 was determined using the Bio-Profil Bio-1D Windows Application V.99.03 program. Three independent experiments were performed.

### Blocking of the expression of Hsp27 and Hsp72 in HeLa and Hep-2 cells

To block the expression of Hsp27 and Hsp72, transfection of HeLa and Hep-2 cells with specific, commercially available siRNAs (Santa Cruz Biotechnology) was performed according to the manufacturer’s protocol. The amounts of specific siRNA and Transfection Reagent were selected experimentally. Our investigations revealed that when the quantity of each component exceeded 4 μl it was cytotoxic to HeLa and Hep-2 cell lines. The blocking procedure was performed in 6-well tissue culture plates at cell density of 2 × 10^5^. Prior to transfection, a mixture containing specific siRNA (4 μl) and Transfection Reagent (4 μl) was prepared and left at room temperature for 45 min. During this time, the medium was removed from the above-mentioned cells and the cells were washed once with 2 ml of the Transfection Medium containing no serum or antibiotics. Next, the previously prepared transfection mixture was added to the cell culture and incubated for 5 h at 37 °C. After this time, 1 ml of growth medium containing serum and antibiotics at a two-fold higher concentration than normal was added without removing the transfection mixture and incubated for the next 24 h at 37 °C. After the incubation, the whole mixture was replaced with fresh normal growth medium and quercetin and imperatorin were added to the cells at appropriate concentrations for 24 h. Additionally negative controls of Transfection Reagent introductoring siRNA into cells and specific siRNAs blocking the expression of Hsps in cells were performed. The effectiveness of the blocking of Hsp27 and Hsp72 gene expression was assessed by immunoblotting. Three independent experiments were performed.

### Caspase activity assay

HeLa and Hep-2 cells were cultured in 6-well plates at the density 2 × 10^5 ^cells/well and treated with quercetin (50 μM) and imperatorin (50 μM) administered separately or simultaneously. For detection of activity of caspase-3, -8, and -9, a commercially available assay kit SensoLyte^®^ AMC Caspase Substrate Sampler Kit Fluorimetric (AnaSpec) was used according to the manufacturer’s protocol. The intensity of fluorescence was measured by a Perkin Elmer 2030 Multilabel Reader VICTOR™ ×4 at *E*_x_/*E*_m_ = 354/442 nm. Each experiment was performed in triplicate.

### Statistical analysis

The quantitative data were expressed as the mean ± standard deviation (SD). All statistical analyses of the data were performed using one-way ANOVA tests followed by a Dunett’s multiple comparison test. Differences with *p* values of < 0.05 were considered statistically significant.

## Results

### The effect of quercetin and imperatorin on cell viability (MTT assay)

The cytotoxic activity of querectin and imperatorin on the viability of HeLa and Hep-2 cell was examined in the presence of different concentrations of both tested compounds applied singly or in various combinations.

### Quercetin

**Fig. 1:**
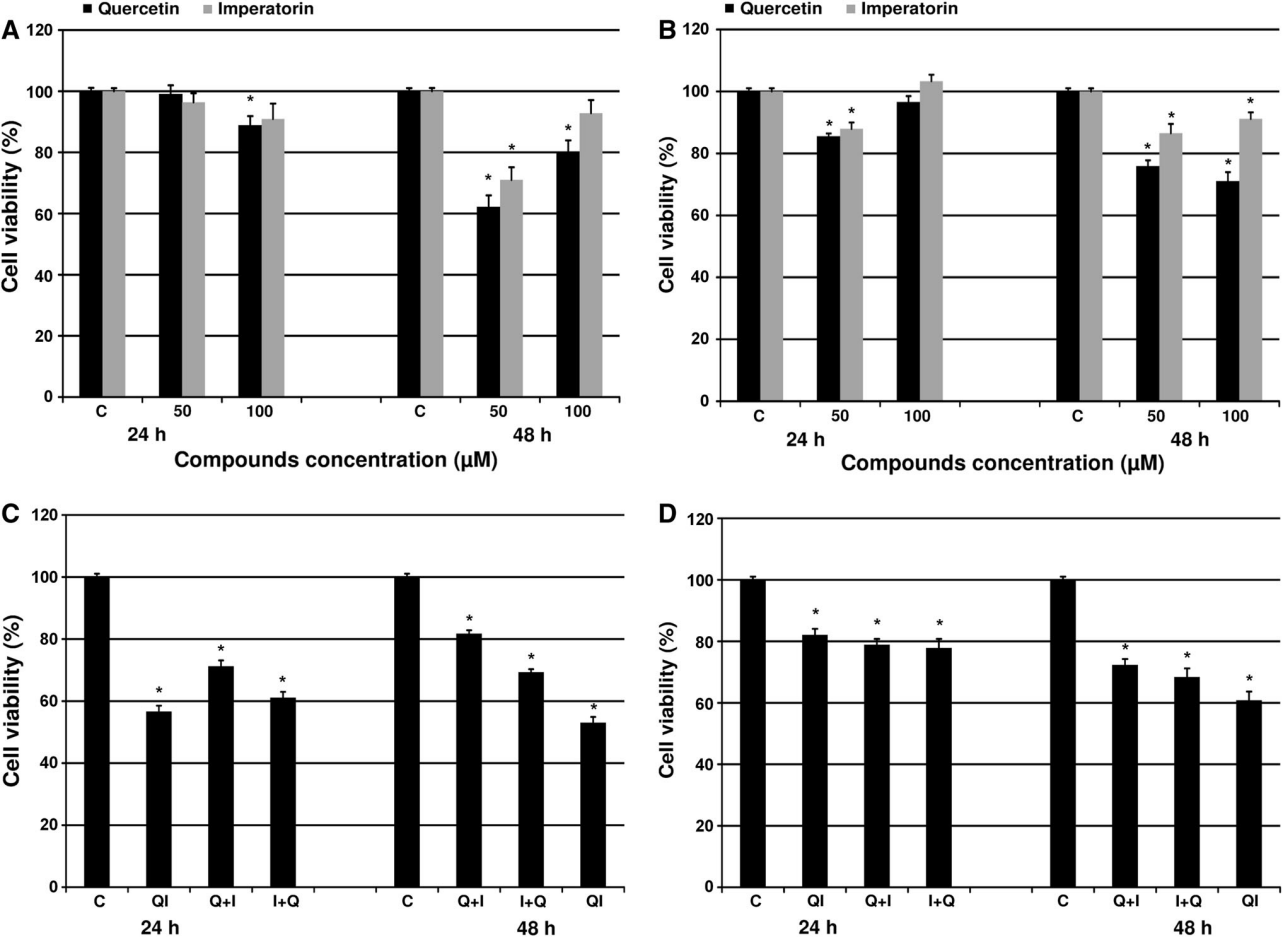
The cytotoxic effect of quercetin, imperatorin (**A**, **B**) and combined treatment with both drugs (**C**, **D**) on viability of HeLa (**A**, **C**) and Hep-2 (**B**, **D**) cells. *C* control, *Q* quercetin, *I* imperatorin, *Q* + *I* cells pre-incubated with quercetin followed by imperatorin administration, *I* + *Q* cells pre-incubated with imperatorin followed by quercetin administration, *QI* cells treated with both drugs administered simultaneously; *p < 0.05

The experiments, we conducted confirmed that 48-h incubations with quercetin markedly diminished HeLa and Hep-2 cell viability in comparison to 24-h incubations (Fig. 1A, B). Exposure of HeLa cells to the 50 μM concentration of flavonoid for 48 h significantly reduced cell viability to about 60 %, while addition of 100 μM decreased viability only by 20 % (Fig. 1A). We achieved similar results in the case of Hep-2 cell line. Treatment of Hep-2 cells for 48 h with culture medium containing quercetin resulted in a strong cytotoxic effect inducing death of 24.6 % cells after incubation with 50 μM of the drug and 29.1 % after application of 100 μM (Fig. 1B). As can be seen in Fig. 1A and B the 24-h incubations with the flavonoid had much weaker effect on HeLa and Hep-2 cell survival.

### Imperatorin

The experiments that we preformed revealed that imperatorin occurred to be less cytotoxic than quercetin to both tested cell lines. Figure 1A clearly indicates on significant reduction of HeLa cell viability to 70.99 % following administration of 50 μM of imperatorin for 48 h, while addition of 100 μM reversed this effect. The MTT viability test revealed no significant effect of imperatorin on HeLa cell survival during 24-h incubations. Exposure of Hep-2 cells to imperatorin treatment demonstrated a cytotoxic effect on cell viability generating a statistically significant percentage of dead cells after both 24 and 48-h incubation (Fig. 1B). However, only in the case of application of 100 μM of imperatorin for 24 h to the culture medium a slight increase in Hep-2 cell viability was observed.

### Combined treatment with quercetin and imperatorin

To estimate the combined effect of the tested compounds on HeLa and Hep-2 cell viability, two variants of the experiment were performed: (1) the cells were pre-incubated with quercetin or imperatorin for 6 or 24 h followed by administration of the other compound for the next 18 or 24 h, and (2) the cells were treated with both drugs simultaneously for 24 and 48 h. These incubation variants were also used in further experiments.

MTT assays revealed that combined treatment with quercetin and imperatorin had a much stronger cytotoxic effect reducing cell viability of both HeLa and Hep-2 cell lines than incubation with single drugs. The 24-h incubation provoked the greatest decrease in HeLa cell viability reaching maximum 43.52 % of dead cells after preincubation with quercetin (Fig. 1C). The other combination variants of 24-h incubations displayed slightly weaker, but still statistically significant reduction of cell viability. The inverse relationship was noticed in the case of 48-h incubations. The pretreatment variants resulted in a clear cytotoxic effect of both drugs on HeLa cells inducing ca. 20 % of dead cells after pretreatment with quercetin, and ca. 30 % as a result of pretreatment with imperatorin (Fig. 1C). However, simultaneous administration of quercetin and imperatorin for 48 h led to progressive reduction of HeLa cell viability to 52.86 %. As shown in Fig. 1D, the decrease in Hep-2 cell viability after 24-h incubation was comparable in all incubation variants and fluctuated around 20–30 % of dead cells. Exposure of the Hep-2 cells to 48-h treatment of both drugs combination diminished cell viability gradually, consequently inducing 39.34 % of dead cells after incubation wit quercetin and imperatorin administered at the same time (Fig. 1D).

### Sensitivity of HeLa and Hep-2 cells to quercetin and imperatorin treatment

Apoptosis, autophagy, and additionally necrosis assays were performed on the HeLa and Hep-2 cell lines in the presence of various concentrations of quercetin and imperatorin administered separately or in different combination variants.

### Quercetin

**Fig. 2:**
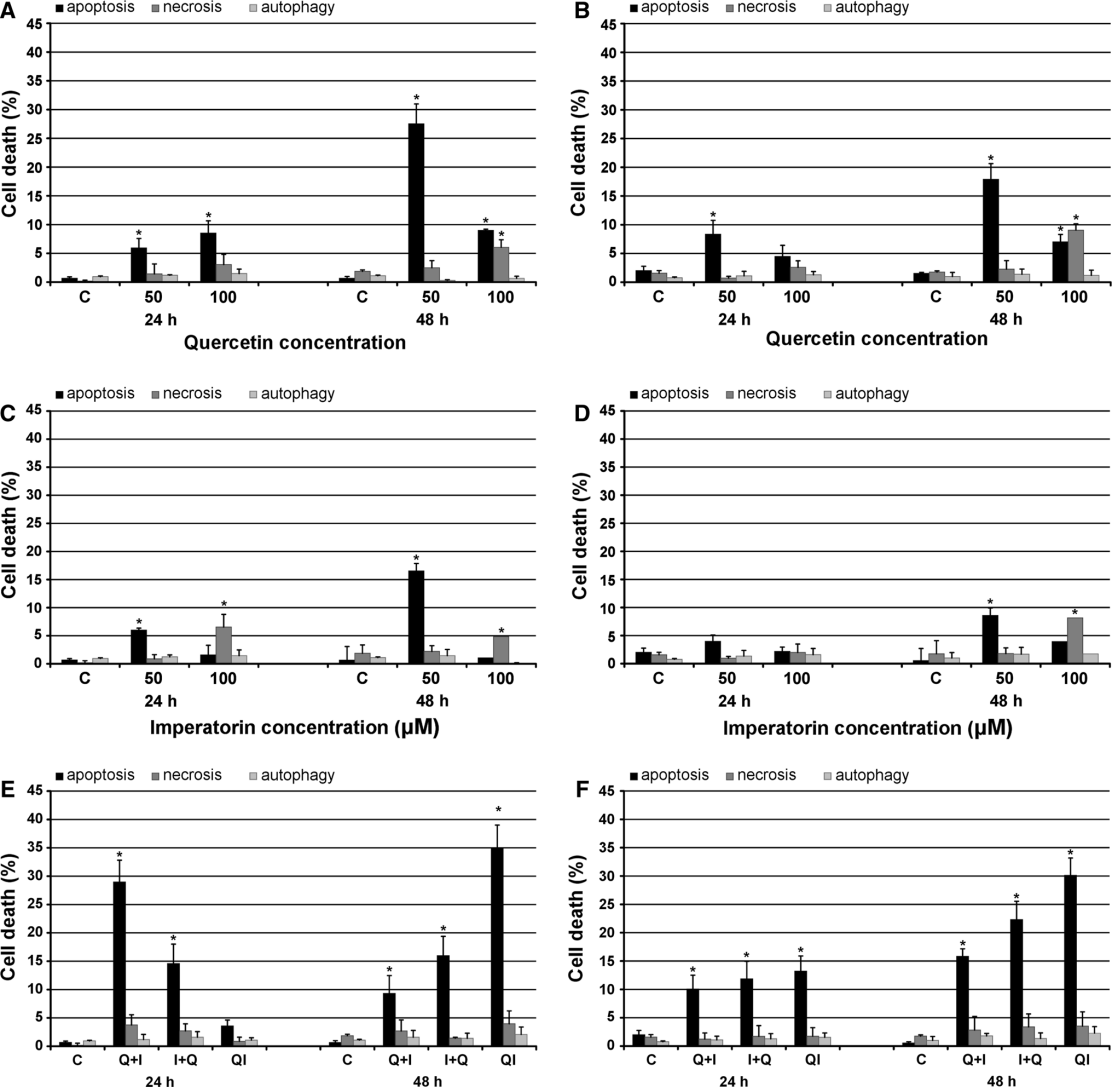
The effect of quercetin (**A**, **B**), imperatorin (**C**, **D**) and combined treatment with both drugs (**E**, **F**) on apoptosis, necrosis, and autophagy induction in HeLa (**A**, **C**, **E**) and Hep-2 (**B**, **D**, **F**) cell lines. *C* control, *Q* quercetin, *I* imperatorin, *Q* + *I* cells pre-incubated with quercetin followed by imperatorin administration; *I* + *Q* cells pre-incubated with imperatorin followed by quercetin administration, *QI* cells treated with both drugs administered simultaneously; *p < 0.05

The HeLa and Hep-2 cells were incubated with quercetin at the concentrations 50 and 100 μM for 24 and 48 h. As can be seen in Fig. 2A, the 48-h incubation of the HeLa cells with the flavonoid resulted in effective apoptosis induction and the highest percentage of apoptotic cells (27.54 %) was noticed after the treatment with 50 μM of the drug, while the number of necrotic cells reached about 2.5 %. In turn, incubation with a 100 μM concentration of quercetin proved to be less effective and the level of apoptotic cells fluctuated around 9.5 %. Furthermore, at the same time an increased level of necrosis was observed (6.06 %). Thus, the 50 μM concentration of quercetin was chosen for further experiments. The 24-h incubation with quercetin was less efficient and initiated apoptosis at a level not exceeding 9 % (Fig. 2A). Treatment with quercetin at both concentrations tested and the incubation time did not stimulate autophagy induction in HeLa cells.

Similar results were obtained in the case of Hep-2 cells. Quercetin at the 50 μM concentration administered to the culture medium for 48 h yielded the highest percentage of apoptotic cells (17.89 %), and the level of necrosis was compared to the control cells (Fig. 2B). Treatment with 100 μM of the flavonoid stimulated apoptosis at a level not exceeding 8 %. However, this concentration exerted a strong cytotoxic effect (9.06 %), and a high percentage of detached cells was observed. Therefore, the concentration of 50 μM of quercetin was used for subsequent investigations. The 24-h incubations with the flavonoid were less effective, and the level of apoptosis reached only about 8.5 % after the treatment with 50 μM of compound. As can be seen in Fig. 2B, quercetin did not appear to be a potent autophagy inducer in Hep-2 cells. The percentage of cells undergoing autophagy was comparable to that in the control.

### Imperatorin

Imperatorin, as well as quercetin, at the 50 μM concentration applied to the culture medium for 48 h appeared to be a potent apoptosis inducer in HeLa cells initiating 16.52 % of apoptotic cells (Fig. 2C). This was accompanied with a low level of necrosis (2.19 %), comparable to that found in the control cells. Treatment with 100 μM of compound had no effect on apoptosis initiation, but an increased number of necrotic cells was noticed. Thus, the 50 μM concentration was chosen for further experiments. The 24-h long incubations generated apoptosis less effectively and the level of apoptotic cells reached only 5.99 % after the treatment with 50 μM of the compound. Moreover, an increasing number of necrotic cells, reaching 6.53 % was observed as a result of incubation with 100 μM of imperatorin. The incubations indicated that imperatorin, likewise quercetin, had no significant influence on autophagy induction in HeLa cells.

Microscopic observations revealed that the treatment with 50 μM of imperatorin for 48 h induced the highest percentage of apoptotic cells (8.58 %) in the Hep-2 cell line (Fig. 2D). Incubation with 100 μM of the compound provoked less effective initiation of apoptosis (3.93 %), causing necrosis in 8.14 % of cells. Incubation of Hep-2 cells with both tested concentrations of imperatorin for 24 h stimulated apoptosis at a level similar to that in the control cells. As can be seen in Fig. 2D, imperatorin was not a potent autophagy initiator in Hep-2 cells.

### Combined quercetin and imperatorin treatment

As shown in Fig. 2E and F, apoptosis induction in both cell lines was dependent on time and the drug application procedure. In the case of the HeLa cell line, we observed that the 48-h incubation proved to be the most effective in apoptosis induction resulting in maximum 34.89 % of apoptotic cells after simultaneous administration of quercetin and imperatorin (Fig. 2E). This was accompanied with a low level of necrotic cells, not exceeding 4 %. Initiation of apoptosis in HeLa cells pre-incubated with imperatorin was less effective, reaching 15.96 % of cells, and the number of necrotic cells was comparable to that in the control. In turn, pretreatment with quercetin was less efficient in apoptosis initiation, but the level of apoptotic cells (9.32 %) was still higher than that observed in the control. As can be seen in Fig. 2E, the 24-h incubation resulted in weaker but statistically significant initiation of apoptosis. The best result was achieved after pretreatment with quercetin, yielding maximum 28.97 % of apoptotic cells. Surprisingly, simultaneous administration of both tested compounds to the HeLa cells culture medium resulted in unexpectedly low percentage of apoptotic cells not exceeding 5 %. The combined drug treatment in all the incubation variants had no effect on autophagy induction in HeLa cells.

In the case of Hep-2 cell line, the strongest initiation of apoptosis was noticed also after the 48-h incubation. The highest level of apoptotic cells reaching 30.13 % was observed after incubation with quercetin and imperatorin applied to the culture medium at the same time (Fig. 2F). This incubation variant also generated a low level of necrotic cells (3.53 %). The other options of the 48-h treatment initiated apoptosis less efficiently, but the level of apoptosis was still significantly higher than that observed in the control cells. The shorter incubation time appeared to be remarkably weaker in apoptosis induction (Fig. 2F). The highest percentage of apoptotic cells reached only 13.22 % after the treatment with quercetin and imperatorin administered simultaneously. Hep-2 cells displayed no sensitivity to autophagy induction upon the tested compounds.

### The effect of quercetin and imperatorin on the expression of cell death marker proteins

#### Activity of caspase-3, caspase-8 and caspase-9

**Fig. 3:**
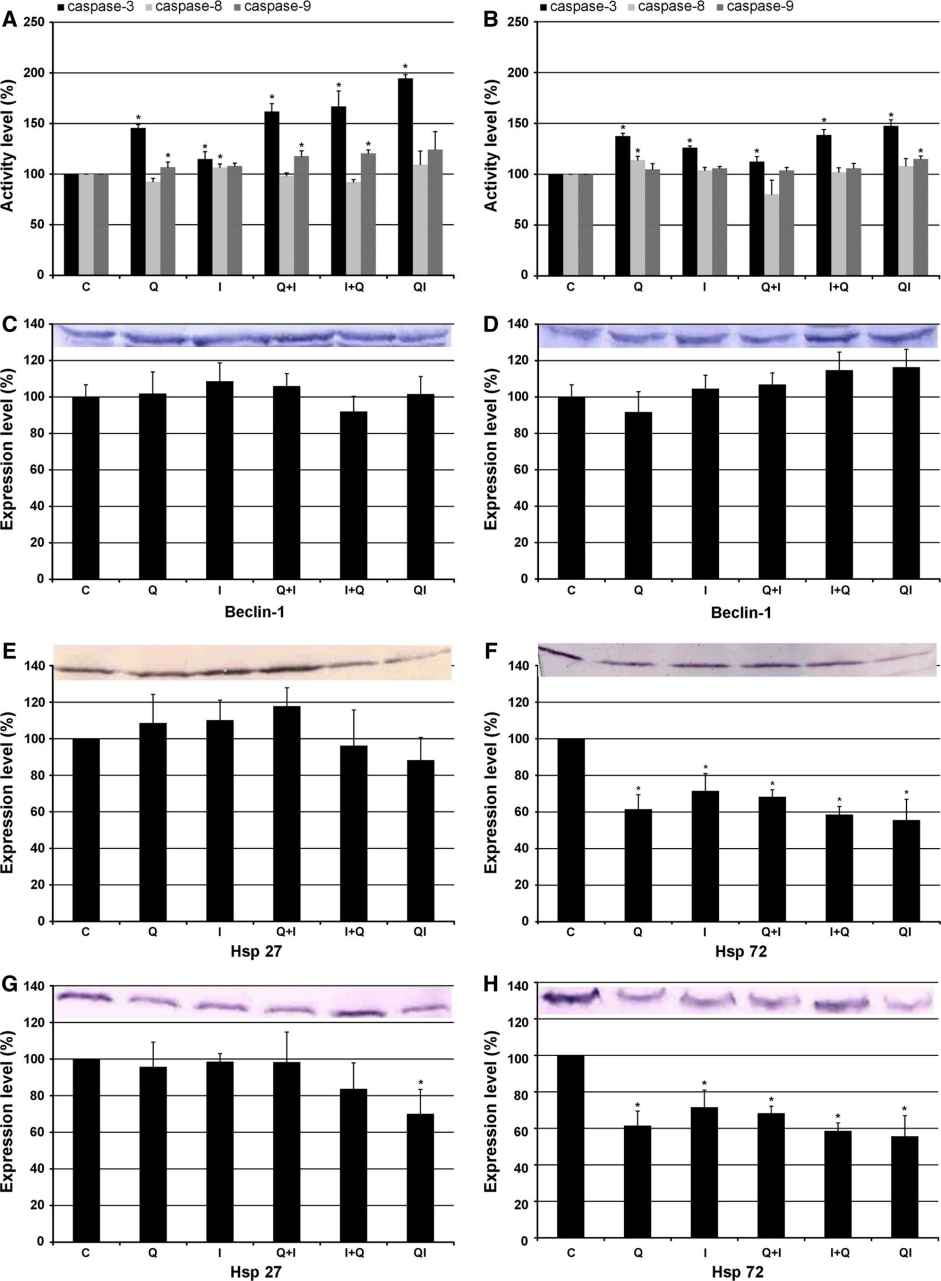
The effect of quercetin (50 μM) and imperatorin (50 μM) administered separately or simultaneously on the activation of caspases (**A**, **B**), expression of beclin-1 (**C**, **D**), Hsp27 (**E**, **G**), and Hsp72 (**F**, **H**) in HeLa (**A**, **C**, **E**, **F**) and Hep-2 (**B**, **D**, **G**, **H**) cell lines. *C* control, *Q* quercetin, *I* imperatorin, *Q* + *I* cells pre-incubated with quercetin followed by imperatorin administration; *I* + *Q* cells pre-incubated with imperatorin followed by quercetin administration, *QI* cells treated with both drugs administered simultaneously. Representative western blots are included; *p < 0.05

The best-studied mediators of apoptosis include caspases, i.e., aspartate-specific cysteine proteases, which function as ultimate effectors during apoptosis. Our investigations carried out on the HeLa cell line revealed a strong increase in the activity of caspase-3 in all experimental variants (Fig. 3A). The best results were achieved after the treatment with quercetin and imperatorin applied to the culture medium simultaneously. This combination of the drugs caused almost a double increase in caspase-3 activity, reaching 194.55 %. In turn, the activity of caspase-8 increased only as a result of incubation with imperatorin (106.6 %). The activation of caspase-9 was observed after incubation with imperatorin and pretreatment variants.

In the case of the Hep-2 cell line, we observed that activation of all caspases was significant weaker (Fig. 3B). Incubation with quercetin and imperatorin applied simultaneously revealed the strongest activation of caspase-3, reaching 147.57 %. The other experimental variants displayed less effective level of caspase-3 activity, but still significant. As shown in Fig. 3B, only incubation with quercetin resulted in activation of caspase-8 (113.9 %). The activation of caspase-9 increased only after the treatment with both drugs administered simultaneously (115.06 %).

### Estimation of beclin-1expression

Beclin-1, one of the Bcl-2 family members, is one of the best-studied autophagy mediators. This protein recruits numerous key autophagic proteins required for proper initiation of autophagosome formation. Cell staining with acridine orange revealed that quercetin and imperatorin turned out not to be potent autophagy inducers in HeLa and Hep-2 cells. These results were further confirmed by molecular analysis, which showed that neither of the tested compounds administered separately or in combination had the ability to initiate autophagy in HeLa (Fig. 3C) and Hep-2 (Fig. 3D) cells.

### Expression of Hsp27 and Hsp72 proteins

The expression of Hsp27 in HeLa cells was dependent on the drug application procedure. Incubation of HeLa cells with quercetin and imperatorin in all experimental variants displayed differences in the expression of Hsp27, however, they were not statistically significant (Fig. 3E). Surprisingly, in the case of Hsp72 expression, we observed an inhibitory effect of the tested compounds in all the experimental options (Fig. 3F). Quercetin and imperatorin administered simultaneously resulted in the strongest reduction in the level of the Hsp72 reaching over 50 %.

The experiments conducted on the Hep-2 cell line revealed that both drugs exerted a strong inhibitory effect on Hsp27 expression, but only as a result of simultaneous administration to the culture medium (Fig. 3G). This drug combination resulted in a decrease in the level of Hsp27 to 69.96 %. Our experiments also demonstrated that both drugs applied separately as well as in combinations lead to a potent reduction of the Hsp72 level. As can be seen in Fig. 3H, the treatment with quercetin and imperatorin given at the same time proved to be the most powerful variant yielding almost two-fold lower expression of Hsp72 (55.55 %).

### Effects of quercetin and imperatorin on the Hsp27 and Hsp72 expression in transfected HeLa and Hep-2 cells

**Fig. 4:**
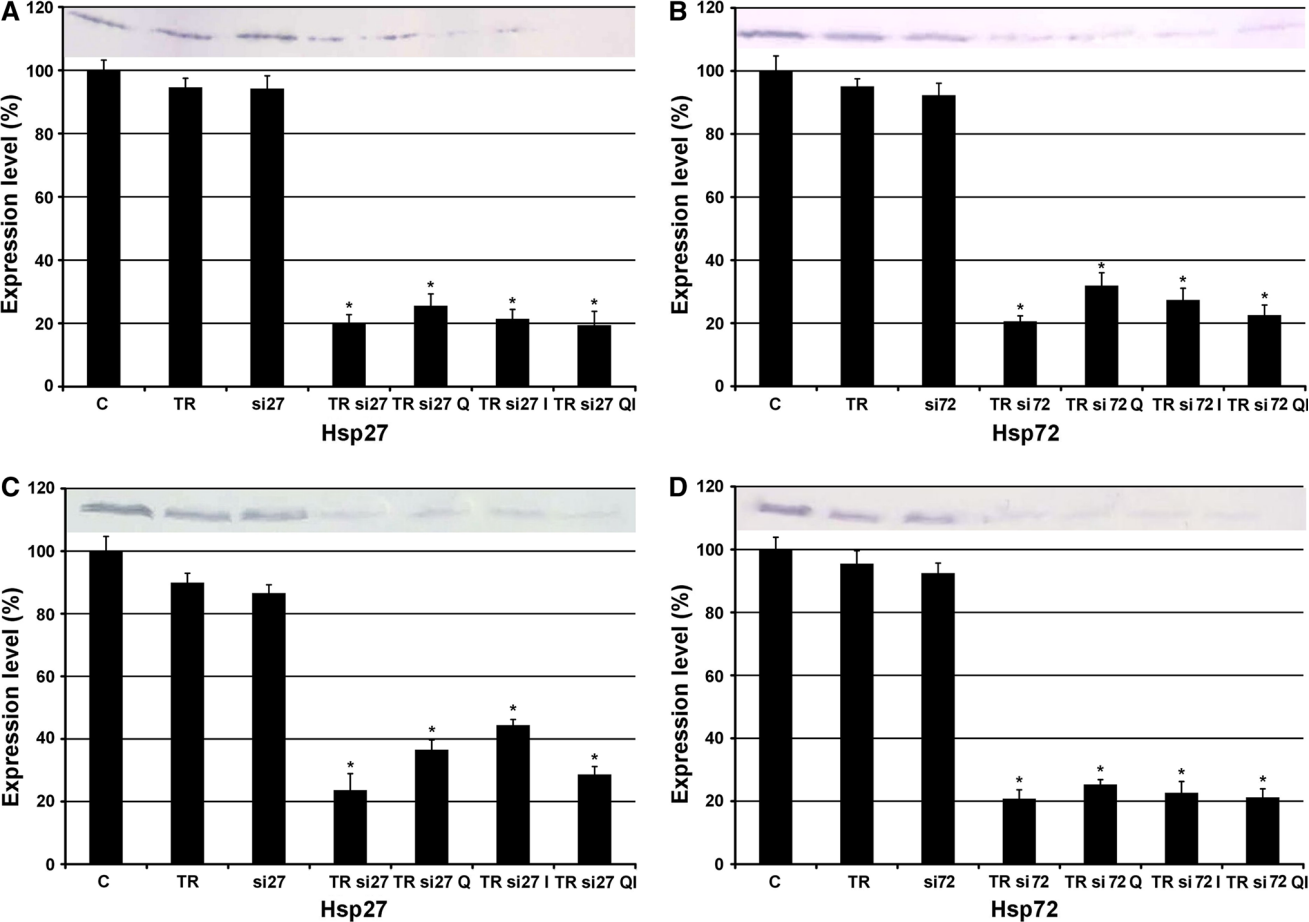
The expression of Hsp27 (**A**, **C**), Hsp72 (**B**, **D**) in transfected HeLa (**A**, **B**) and Hep-2 (**C**, **D**) cell lines subsequently treated with quercetin (50 μM) and imperatorin (50 μM) for 24 h. *C* control; *TR* HeLa/Hep-2 cells incubated only with Transfection Reagent (TR) introductoring siRNA into cells; *si27/si72* HeLa/Hep-2 cells incubated only with specific siRNA blocking the expression of Hsp27 or Hsp72 in cells; *TRsi27/TRsi72* HeLa/Hep-2 cells incubated with complex of TR and specific siRNA which blocks the expression of Hsp27 or Hsp72 in cells; *Q* quercetin; *I* imperatorin; *QI* drugs administered simultaneously. Representative western blots are included; *p < 0.05

The efficiency of blocking the expression of Hsp27 and Hsp72 genes with specific siRNA was examined by immunoblotting analysis. Our experiments demonstrated that transfection of HeLa and Hep-2 cells proved to be successful and remarkably diminished the level of Hsp27 and Hsp72 expression. Transfected HeLa cells showed a marked reduction of the Hsp27 level after the treatment with quercetin and imperatorin administered simultaneously, which reduced the protein expression to 19.3 % (Fig. 4A). Both drugs applied at the same time also appeared to be the most efficient combination inhibiting the expression of Hsp72 to 22.45 % (Fig. 4B). Although administration of quercetin and imperatorin separately resulted in a slight increase in the expression of both proteins in transfected HeLa cells, the levels of Hsp27 and Hsp72 were still strongly reduced by over 60 %.

Similar results were achieved in the case of transfected Hep-2 cells. Our experiments demonstrated that the most effective reduction of Hsp27 and Hsp72 level was noticed after the simultaneous treatment with quercetin and imperatorin. The expression of Hsp27 was reduced to 28.53 % (Fig. 4C), while Hsp72 expression reached only 21.09 % (Fig. 4D), respectively. Despite blocking the genes of Hsp27 and Hsp72 expression, a minor increase in their level after incubation with the drugs applied separately was observed. However, the expression of both proteins was still strongly diminished by more than 50 %.

### Sensitivity of transfected cells on apoptosis, autophagy, and necrosis induction upon quercetin and imperatorin

**Fig. 5:**
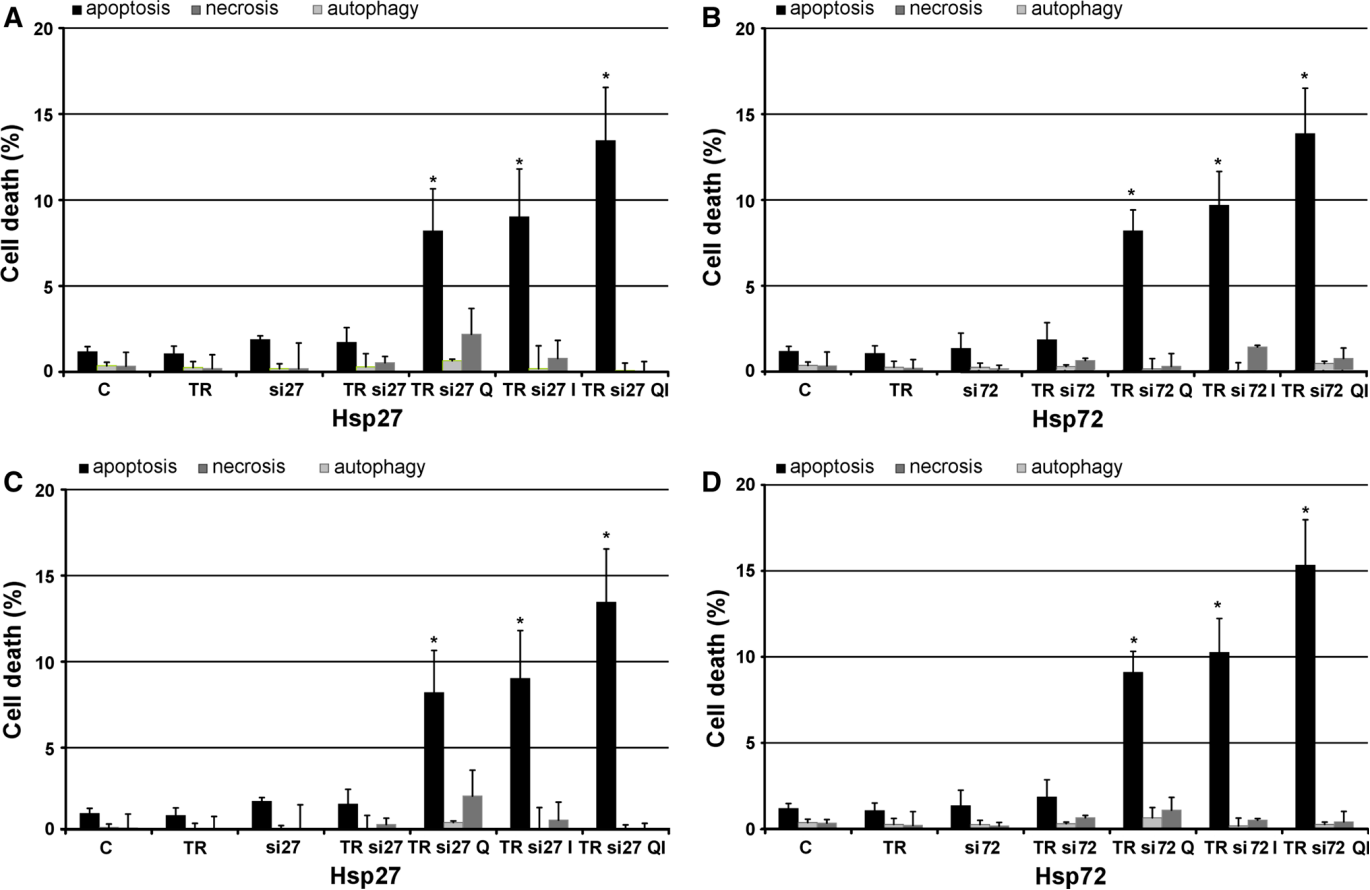
The level of apoptosis, necrosis, and autophagy induction in HeLa (**A**, **B**) and Hep-2 (**C**, **D**) cells after blocking the Hsp27 (**A**, **C**) and Hsp72 (**B**, **D**) expression subsequently treated with quercetin (50 μM) and imperatorin (50 μM) for 24 h. *C* control; *TR* HeLa/Hep-2 cells incubated only with Transfection Reagent (TR) introductoring siRNA into cells; *si27/si72* HeLa/Hep-2 cells incubated only with specific siRNA blocking the expression of Hsp27 or Hsp72 in cells; *TRsi27/TRsi72* HeLa/Hep-2 cells incubated with complex of TR and specific siRNA which blocks the expression of Hsp27 or Hsp72 in cells; *Q* quercetin; *I* imperatorin; *QI* drugs administered simultaneously; *p < 0.05

The transfected HeLa and Hep-2 cells were exposed to quercetin and/or imperatorin treatment. The experiments conducted revealed that the silencing of Hsp27 and Hsp72 gene expression was strictly correlated with the increased sensitivity of HeLa (Fig. 5A, B) and Hep-2 (Fig. 5C, D) cells to apoptosis induction by the tested drugs. It should be noted that blocking of the gene expression of Hsp27 and Hsp72 in both cell lines caused such strong sensitization of cancer cells that extending the incubation time to 48 h resulted in a strong cytotoxic effect. We observed extremely sparse cell cultures and a high percentage of detached cells (data not shown).

Morphological analysis performed after the 24-h incubation of HeLa cells with diminished Hsp27 and with diminished Hsp72 expression revealed effective induction of apoptosis, especially after the simultaneous treatment with quercetin and imperatorin (Fig. 5A, B). The other incubation variants resulted in a lower percentage of dead cells, but still higher that than observed in the control. No significant effects in necrosis induction were observed. Furthermore, downregulation of Hsp27 and Hsp72 gene expression had no impact on autophagy induction in the HeLa cells.

Microscopic observations after the 24-h incubation of Hep-2 cells with decreased expression of Hsp27 and Hsp72 demonstrated an increased number of apoptotic cells especially after the treatment with quercetin and imperatorin administered at the same time (Fig. 5C, D). The other experimental variants were less effective in apoptosis induction, but still the results were significant. Silencing of the Hsp27 and Hsp72 gene expression in Hep-2 cells had no effect on necrosis induction or initiation of autophagy.

Additional experiments with negative controls of Transfection Reagent and siRNAs that we performed demonstrated no effect of these factors on cell death induction.

## Discussion

Elimination of cancer cells from human organism is a real challenge for scientists all over the world. The complexity of the nature of cancer cells still makes it the focus of attention of many researches. A body of literature describes cancer cells characterized by altered metabolism, pH imbalances, hypoxia, and relatively undamaged DNA synthesis checkpoints leading to heavy mutation load. Additionally, cancer cells often overexpress Hsps, probably because of the stressful conditions in which tumors reside, but also because of the benefits of Hsps cytoprotection [33, 34]. Recently, more attention has been paid to the phenomenon of Hsp overexpression in cancer cells, among which Hsp27 and Hsp72 have been attached a great deal of importance as apoptotic chaperone proteins. Therefore, these proteins are one of the key elements blocking the programmed cell death induction, consequently leading to the reduction of cancer cells sensitivity to commonly used chemotherapy [14].

In the past years, quercetin has appeared as a potential therapeutic agent for treatment of various types of cancers due to its ability of apoptosis induction. The proapoptotic activity of quercetin against cancer cells has been well documented, e.g., in leukemia (HL-60) [35], osteosarcoma (MG-63) [36], colon (LoVo) [37], breast (MCF-7, U937) [37–39], brain (MOGGCCM, T98G) [40–42], and lung (NCI-H209) [43] cell lines. Since quercetin has been examined in a variety of cancer cells, it is important to understand the effect of the flavonoid also on normal, non-malignant cells. Several lines of evidence indicates that quercetin exerts a selective cytotoxic effect on both normal and transformed cells. Studies conducted on non-malignant cells such as neurons [44], mouse thymocytes [45], and bone marrow cells [46] proved the beneficial cytoprotective effects of quercetin associated with free radicals scavenging and modulating the biochemical markers of oxidative stress and antioxidant enzymes [47, 48].

Several reports have proposed the mechanisms involved in the proapoptotic properties of this flavonoid consisting in activation of kinases, downregulation of survival pathways, mitochondrial potential perturbation, release of cytochrome *c*, and, in consequence, activation of caspase-3 and caspase-9 [18, 22, 35, 36, 38]. Experiments have shown that micromolar concentrations of quercetin in the range of 29–150 μmol/l can induce apoptosis. It has also been reported that quercetin has an effective ability to inhibit Hsps synthesis. Literature data suggest that it blocks the expression of Hsp27 and Hsp72 at the transcriptional level by preventing binding of heat shock factor 1 and 2 (Hsf1, Hsf2) to the conserved DNA sequence called the heat shock element (HSE). Another theory assumes that quercetin blocks additional modifications needed to activate Hsf, such as post-translational phosphorylation [17, 21].

Assessing cell death has become an indispensable part of biological research, with novel unique technologies being released at a rapid rate. One of the measurement with a long-held reputation as the conventional cell proliferation assay is MTT, a simply preliminary test, used for measuring cell viability and proliferation of living cells and evaluation of the cytotoxicity and effectiveness of a potential drug candidate. MTT test, supported by a strong body of literature, is a metabolic activity assay which involves the use of tetrazolium salt cleaved in the mitochondria of metabolically active cells to colored formazan salt that can be measured by absorbance. The amount of formazan dye produced is directly proportional to the quantity of metabolically active cells [49–51].

The present study indicates quercetin as an effective antiproliferative drug. The experiment that we performed demonstrated a significant reduction of the viability of both cell lines studied especially after 48 h of treatment with 50 μM of quercetin yielding 37.97 % of dead cells in the case of the HeLa cell line and 29.1 % in the case of the Hep-2 cell line.

Since the MTT test revealed cell death in both tested cell lines, we used a staining method with specific fluorochromes to determine the types of cell death. Induction of apoptosis and necrosis in HeLa and Hep-2 cells upon quercetin treatment was assayed by simultaneous use of the double Hoechst 33342/PI staining method. This convenient and rapid measurement is commonly used to distinguish normal, apoptotic, and necrotic cells. The blue fluorescent DNA stain Hoechst is a cell permeable nucleic acid that binds within the minor groove of double-stranded AT-rich regions in DNA. Thus, it allows to identify chromatin condensation and fragmentation by staining the condensed nuclei of apoptotic cells. In turn, the red-fluorescent PI is a cell impairment DNA dye, which only penetrates the cells with the lost cellular membrane integrity [52, 53].

The morphological analysis, we performed and confirmed that quercetin was an effective apoptosis inducer in HeLa and Hep-2 cells at the 50 μM concentration after 48 h of the treatment, yielding the maximum percentage of dead cells 25.54 % in the HeLa cell line and 17.89 % in the case of the Hep-2 cell line, respectively. At the molecular level, the apoptosis induction in the HeLa and Hep-2 cells was closely correlated with the expression of Hsp27 and Hsp72 protein, well-known apoptosis inhibitors. Our experiments demonstrated an effective inhibitory effect of the flavonoid on Hsp72 expression in both cell lines studied, resulting in sensitizing HeLa and Hep-2 cells to apoptosis initiation. These results corresponded with an increased activity of caspase-3 in both cell lines and caspase-8 only in Hep-2 cells after the quercetin treatment. Based on these results, it can be suggested that the pathway of the apoptotic signal depended on the cell line. Furthermore, we can hypothesize that apoptosis induced by the flavonoid may proceed not only through the mitochondrial pathway.

Several articles have reported that quercetin has the ability to trigger autophagy in cancer cells [54]. Paradoxically, the morphological analysis carried out revealed that the flavonoid was a weak autophagy inducer in both HeLa and Hep-2 cell lines. These results were further verified at the molecular level by measurement of the beclin-1 level, which showed no significant changes in its expression. Thus, quercetin has no ability to induce autophagy in HeLa and Hep-2 cells.

An increasing number of reports has described imperatorin as a compound with potent anticancer properties manifested in inhibition of cell proliferation and induction of apoptosis in several types of cancer, i.e., hepatoma (HepG2) [55] and leukemia (HL-60) [56]. The results of various biochemical analysis revealed the ability of imperatorin to arrest cell cycle and trigger apoptosis via both death receptor and mitochondria mediated pathways. However, the underlying mechanism of this proapoptotic activity is not yet fully understood [23, 27, 55, 56]. Despite the fact that imperatorin, widely used in ethnomedicine for many centuries, has been the subject of intensive research for only a few years, there is still no literature data indicating toxicity of imperatorin to normal cells.

Our experiments on HeLa and Hep-2 cell lines demonstrated that imperatorin likewise quercetin exerted strong antiproliferative effect markedly diminishing viability of both cell lines and inducing 29.01 % of dead HeLa cells and 13.5 % of dead Hep-2 cells after 48-h treatment with the 50 μM concentration. The validity of the results mentioned above was further corroborated by morphological analysis which clearly indicated on imperatorin as an effective apoptosis initiator resulting in maximum 16.52 % of dead cells in the case of HeLa cells and 8.58 % in the case of Hep-2 cells after treatment with 50 μM for 48 h. At the molecular level, this was accompanied with the efficient inhibition of the expression of Hsp72 in both cell lines, which made the cells more sensitive to apoptosis induction. Although currently there is very little understanding about a possible mechanism of Hsp inhibition by imperatorin, our experiments suggest imperatorin as a possible suppressor of Hsp expression. Induction of apoptosis in HeLa and Hep-2 cells upon the imperatorin treatment was additionally confirmed by measurements of caspase activity, which revealed an increasing activity all of the tested caspases in HeLa cells and only caspase-3 in the Hep-2 cell line. These results allow a hypothesis that imperatorin induces apoptosis in HeLa and Hep-2 cells via the mitochondrial and death receptor pathway. On the other hand, imperatorin turned out not to be a potent autophagy inducer in HeLa and Hep-2 cells. Microscopic analysis, as well as measurement of the beclin-1 expression gave no support to challenge this hypothesis.

It is well documented that natural plant extracts and natural compounds exhibit a synergistic anti-tumor effect [57]. Herein, we decided to examine, for the first time, the effect of the combined treatment of quercetin and imperatorin on cell death induction in HeLa and Hep-2 cells. We measured cell death induction in cells pretreated with quercetin or imperatorin followed by imperatorin or quercetin administration and with both compounds applied simultaneously. It is worth noting the different kinetics of action of quercetin and imperatorin in the tested cell lines dependent on time and the drug application procedure. Our experiments demonstrated that the combination of both drugs applied at the same time for 48 h was the most effective in inhibiting of proliferation generating 47.14 % of dead HeLa cells and 39.34 % of dead Hep-2 cells. Verification obtained results by morphological analysis confirmed that simultaneously administration of both drugs tested were the most genuine and powerful drug combination in apoptosis induction, reaching maximum 34.89 % of dead cells in the case of the HeLa cells and 30.13 % in the case of the Hep-2 cell line. Surprising seemed to be a very low level of apoptotic HeLa cells induced as a result of 24 h treatment with both drugs applied at the same time. This can be explained by unspecific reaction of HeLa cells that caused the detachment of cells from the substrate and prevented precise morphological analysis.

Obtained results were consistent at the molecular level with the strongest inhibition of Hsp27 and Hsp72 expression and the intense activation of caspase-3 in both cell lines. The combined treatment with quercetin and imperatorin also contributed to activation of caspase-9 in HeLa and Hep-2 cells. These findings may suggest synergistic action of quercetin and imperatorin, which makes them a novel effective couple inducing apoptosis. Neither drug at all the combination variants had a significant effect on autophagy induction in both cell lines.

Numerous studies indicate that the unusual expression of Hsp27 and Hsp72 is crucial for progression of different types of human cancer and is closely associated with resistance to apoptosis by enhancement of cell growth. It has been reported that Hsp27 and Hsp72 can regulate apoptosis through their ability to interact with the key elements of the apoptotic-signaling pathways. However, the mechanism by which this is achieved still remains unclear. Nevertheless, it is known that overexpression of Hsp27 and Hsp72 in tumor cells negatively regulates apoptosis by preventing recruitment of procaspase-9 and procaspase-3 and sequestration of cytostolic cytochrome *c* from Apaf-1 after its release from the mitochondrion, thereby preventing the assembly of the functional apoptosome. Indirect experimental evidence suggests that downregulation of Hsps expression in cancer cells may either sensitize them to chemotherapy or commit to apoptosis [15, 57–65]. Thus, to obtain direct evidence of the participation of Hsp27 and Hsp72 in resistance to apoptosis of the cancer cells studied, silencing of the Hsp27 and Hsp72 gene expression by specific siRNA was performed. Our experiments at the molecular level demonstrated that the transfection of HeLa and Hep-2 cells occurred to be successful and resulted in marked reduction of the Hsp27 and Hsp72 level in both cell lines, consequently sensitizing HeLa and Hep-2 cells to apoptosis induction. Downregulation of the Hsp27 and Hsp72 expression in HeLa and Hep-2 cells, and further treatment with the tested drugs resulted in an increasing percentage of apoptotic cells. Once again, the combined drug treatment proved to be more efficient in apoptosis induction in both transfected cell lines than application of single agents. Nevertheless, the results mentioned were not as high as those observed in non-transfected cells. The intriguing blocking of Hsp27 and Hsp72 gene expression still had no effect on autophagy induction upon the quercetin and imperatorin treatment.

## Conclusion

In the present study, we have provided evidence supporting the potential of quercetin and imperatorin as effective anticancer compounds inhibiting proliferation and inducing apoptosis in HeLa and Hep-2 cell lines. Herein, we have also demonstrated for the first time that the combined treatment with quercetin and imperatorin administered simultaneously greatly induced growth inhibition in cancer cell and consequently bringing them to apoptotic pathways. Furthermore, we have reported that silencing of Hsp27 and Hsp72 gene expression in HeLa and Hep-2 cells had no significant effect on sensitizing both cell lines on apoptosis induction in comparison to non-transfected cells. This might be explained by incomplete Hsp inhibition additionally hindered by quercetin and imperatorin. Nevertheless, our findings illustrate the potential use of combination quercetin with imperatorin as an effective, novel tool to eliminate various types of cancer. However, further studies are needed to elucidate the precise nature of this phenomenon.
